# Efficacy and safety of patiromer for non-dialysis and dialysis patients with hyperkalemia: the randomized, placebo-controlled and long-term study

**DOI:** 10.1007/s10157-024-02585-5

**Published:** 2024-11-24

**Authors:** Naoki Kashihara, Yasuro Kumeda, Yorihiko Higashino, Yoshitaka Maeda, Yoko Kaneko, Hidetoshi Kanai, Yuko Taniguchi, Takayuki Ishii, Yusuke Tomioka

**Affiliations:** 1 Kawasaki Medical School, Okayama, Japan; 2 Minamiosaka Hospital, Osaka, Japan; 3 Higashi Takarazuka, Satoh Hospital, Hyogo, Japan; 4 JA Toride Medical Center, Ibaraki, Japan; 5 Tsukuba Central Hospital, Ibaraki, Japan; 6 Kokura Memorial Hospital, Fukuoka, Japan; 7 Zeria Pharmaceutical.Co., Ltd, Tokyo, Japan

**Keywords:** Hyperkalemia, Phase II, Placebo-controlled, Long-term safety, Patiromer

## Abstract

**Background:**

The objectives of this phase two study are to investigate the efficacy of two starting doses of 8.4 g and 16.8 g and evaluate the long-term safety of patiromer in Japanese patients with hyperkalemia.

**Methods:**

This study comprised three cohorts; non-dialysis patients with baseline serum potassium (sK) level of 5.1 to < 6.0 mmol/L (NDC1); 6.0 to < 6.5 mmol/L (NDC2); dialysis patients with baseline sK level of 5.5 to < 6.5 mmol/L (DC). The study design was one-week, randomized, double-blind, placebo-controlled, and open label extension for one year in NDC1, open label during the study in NDC2 and DC. Patients were randomly assigned to patiromer 8.4 g, 16.8 g or placebo in NDC1, 8.4 g or 16.8 g in NDC2 and DC. Dose was adjusted up to 25.2 g according to the titration algorism in open label period.

**Results:**

A total of 185 patients were randomized (NDC1:153, NDC2:10, and DC:22). The primary endpoint of the change in least squares mean sK levels at Week 1 in NDC1 was  – 0.55,  – 0.77 and  – 0.10 mmol/L for the 8.4 g, 16.8 g and placebo group (*P* < 0.001 for the patiromer group vs the placebo group). In all cohorts for each patiromer group, more than 80% of patients achieved normal sK at Week 5. There was no severe treatment-related adverse event.

**Conclusion:**

Treatment with patiromer was effective in lowering and maintaining target sK levels, also well tolerated for one year in Japanese patients with hyperkalemia.

**Supplementary Information:**

The online version contains supplementary material available at 10.1007/s10157-024-02585-5.

## Introduction

Hyperkalemia is a serious electrolyte disturbance that can cause life-threatening arrhythmias, cardiac arrest and sudden death [1, 2]. In a Japanese observational study, which assessed the prevalence, treatment pattern, and long-term risk of hyperkalemia in real-world clinical practice, hyperkalemia prevalence was 67.9 per 1000 and increased in patients with chronic kidney disease (CKD), heart failure and renin–angiotensin–aldosterone system inhibitors (RAASi) use. Mortality increased at higher serum potassium (sK) levels and in patients with advanced CKD stages. In addition, more than 50% of patients with hyperkalemia experienced RAASi discontinuation after first hyperkalemia episode [3]. The use of RAASi is recommended by guidelines for CKD and heart failure to reduce disease progression and improve outcomes [4–7]. However, management of hyperkalemia often requires dose reduction or discontinuation of RAASi due to the limited use of traditional treatment options for chronic hyperkalemia [8, 9].

Although sodium polystyrene sulfonate and calcium polystyrene sulfonate are commonly used potassium binders for more than 50 years, these agents are not sufficiently tolerated, and their use occasionally encountered serious side effects, such as bowel necrosis [10, 11]. In fact, some patients were forced to discontinue their use due to patient preferences, treatment burden, the difficulty of intake, sandy texture, dosing frequency, and adverse events [3, 11].

Patiromer is a sodium-free, non-absorbed, high-capacity potassium binding polymer for oral suspension that binds potassium in exchange for calcium throughout the gastrointestinal tract. Patiromer has been approved in the US, Europe and other locations for the treatment of hyperkalemia; the recommended starting dose is 8.4 g administered orally once daily and it is allowed to adjust dose by 8.4 g daily as needed at one-week intervals up to 25.2 g [12, 13].

This phase two study was designed to investigate the efficacy of two starting doses of 8.4 g and 16.8 g, and also to evaluate the long-term safety of patiromer in Japanese patients with hyperkalemia.

## Materials and methods

### Study design

**Fig. 1:**
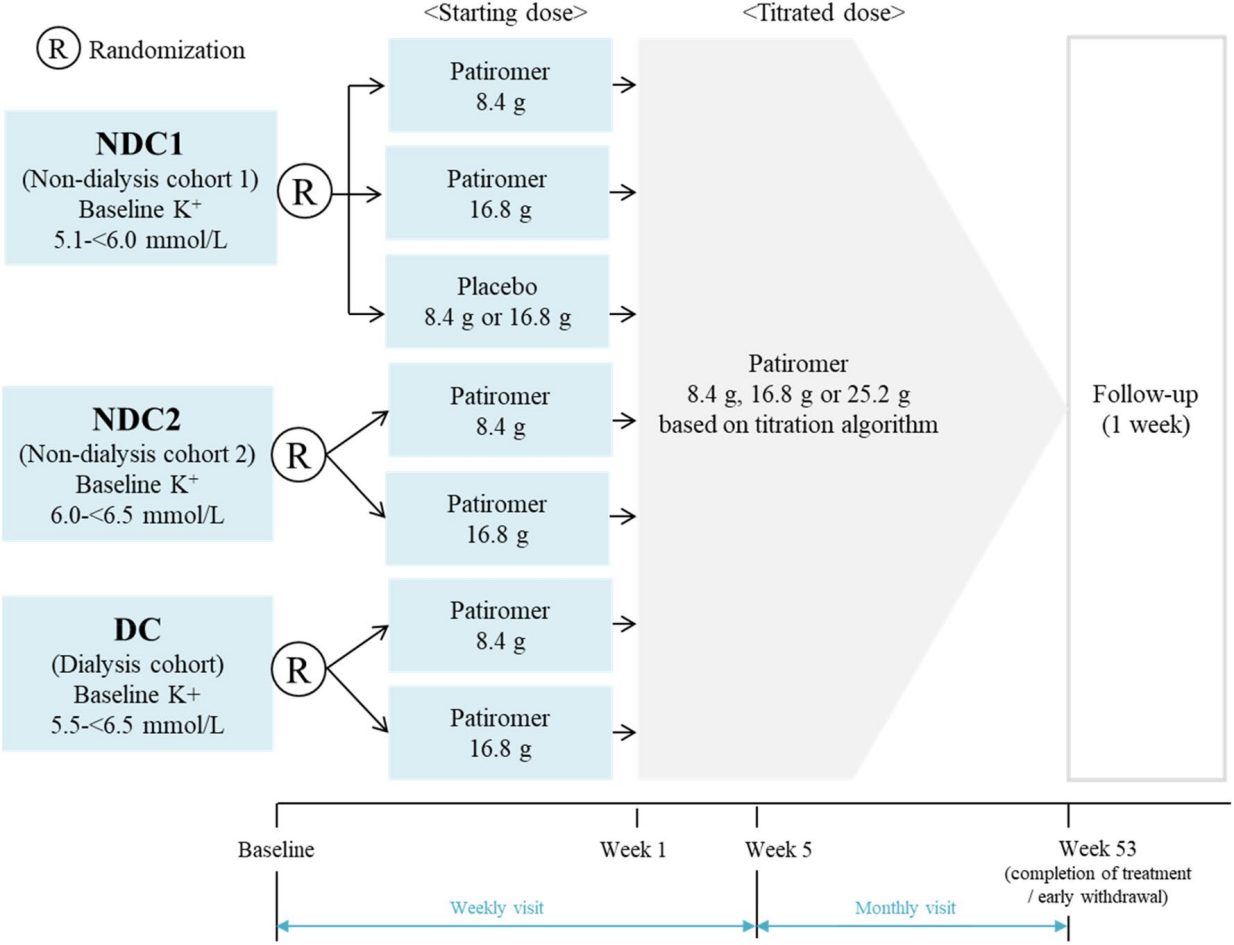
Study design

This was a multicenter, randomized, phase two, long-term study (ClinicalTrials.gov: NCT03799926). The study design is shown in Fig. 1. Three cohorts were defined based on sK level and dialysis use; non-dialysis cohort 1 (NDC1) [non-dialysis patients with baseline sK level of 5.1 to < 6.0 mmol/L], non-dialysis cohort 2 (NDC2) [non-dialysis patients with baseline sK level of 6.0 to < 6.5 mmol/L] and dialysis cohort (DC) [dialysis patients].

In NDC1, the study design was one-week double-blind, comparing pairwise treatment groups between patiromer 8.4 g, 16.8 g and placebo, and open label after Week 1 to Week 53. In NDC2 and DC, the study design was open label during the study.

This study was conducted in Japan between February 2019 and February 2021. The study protocol was approved by the local or central institutional review board for each participating site. Patients were enrolled in this study after their written informed consents were obtained; the study was performed in accordance with the ethical principles of the Declaration of Helsinki and current Good Clinical Practice and in compliance with local regulatory requirements.

### Patients

The eligible participants ranged in age from 20 to 80 years old, with sK levels falling between 5.1 to less than 6.5 mmol/L for non-dialysis patients, and between 5.5 to less than 6.5 mmol/L for dialysis patients. Key-exclusion criteria were hyperkalemia that required emergency intervention, anuria (only for non-dialysis cohort) or history of acute kidney injury in the previous 3 months, severe gastrointestinal disorders and use of sodium polystyrene sulfonate, calcium polystyrene sulfonate, sodium zirconium cyclosilicate, and potassium supplement in the last seven days prior to screening.

### Randomization and treatment

Patients in NDC1 were randomly assigned (1:1:1) to patiromer starting dose of 8.4 g, 16.8 g or placebo using dynamic allocation by the minimization method. The allocation factor was baseline sK level by local measurements 5.1 to < 5.5 mmol/L or 5.5 to < 6.0 mmol/L, and the number of patients in each group was balanced within each site. In NDC1, patients in the placebo group took patiromer after Week 1.

Patients in NDC2 and DC were randomly assigned (1:1) to patiromer starting dose of 8.4 g or 16.8 g, respectively (Supplementary Table 1 for additional details).

Each dose was administered as an oral suspension once a day. If a patient’s sK level (local laboratory) at each visit was outside of a target range (3.8 to < 5.1 mmol/L), patiromer dose was adjusted in increments of ±8.4 g to the individualized effective dose according to a prespecified algorithm. Patiromer dose could be ranged from a minimum of 8.4 g (at or before Week 5) or 0 g (after Week 5), to a maximum of 25.2 g.

### Procedure

Patients visited weekly during the first 5 weeks (Week 1–5) and monthly for the following study period (Week 5–53). Local measurements of sK were used for assessments of eligibility and dose adjustment. Central measurements of sK were used for assessments of efficacy and safety. The concomitant use of the following drugs or treatment was prohibited during the study period: potassium supplements; any treatment of hyperkalemia except for study drug. In NDC1 and NDC2, no new potassium-altering chronic medications such as RAASi, loop and thiazide diuretics, non-selective beta blockers, and SGLT2 inhibitors were to be initiated and doses of these medications were not changed by Week 5. Details regarding the potassium-altering chronic medications are provided in Supplementary Table 2. Patients were observed through the safety follow-up period for one week and the following period until their termination of treatment, including early withdrawal patients.

### Study endpoints

The primary efficacy endpoint was the mean change in sK level from baseline (sK change) at Week 1 in NDC1. The other efficacy endpoints were sK change, proportion of normokalemic patients, time to first normalization of sK, and continuation of RAASi. Normal sK range was 3.8 to < 5.0 mmol/L in NDC1 and NDC2, 3.8 to < 5.5 mmol/L in DC. The safety endpoints were incidence of adverse events and a decrease in at least one sK level < 3.5 mmol/L. Adverse events, laboratory assessments, electrocardiograms and vital signs were summarized descriptively for the entire study.

### Statistical analysis

Efficacy analysis was performed on the full analysis set (FAS) (Supplementary Table 3). Primary endpoint was assessed using an analysis of covariance with each treatment group as explanatory variable and baseline sK as covariate. A contrast test was performed using contrast coefficients of 8.4 g group: 16.8 g group: placebo group = 1:1:-2. Pairwise comparisons between each patiromer group and the placebo group were also performed. For sK change at each visit and the proportion of normokalemic patients under treatment at each visit, summary statistics and 95% CIs were used. For handling individual sK data, central measurement was analyzed. Local measurement was used for analysis in case of missing data. Safety analysis was performed on the safety analysis set (SAF) (Supplementary Table 3).

The sample size was not determined by using statistical method because this study was exploratory. Taking into account for the feasibility of patient recruitment, 50 patients per each treatment group (8.4 g, 16.8 g and placebo) were set as sample size in NDC1. The target number of patients in DC was estimated to be 20 randomized patients. Assuming a 69% completion rate at Week 53, it was estimated that 180 randomized patients would yield 124 evaluable patients to ensure safety data for more than 100 Japanese patients with one-year exposure [14].

All analyses were conducted using SAS version 9.4 (SAS Institute Inc.). The code was broken after all patients in NDC1 completed Week 4 or discontinued the study by Week 4. Subgroup analysis by sex, age, body mass index, primary disease (CKD, diabetes mellitus, heart failure and hypertension), RAASi use and baseline sK (local) was performed.

## Results

**Fig. 2:**
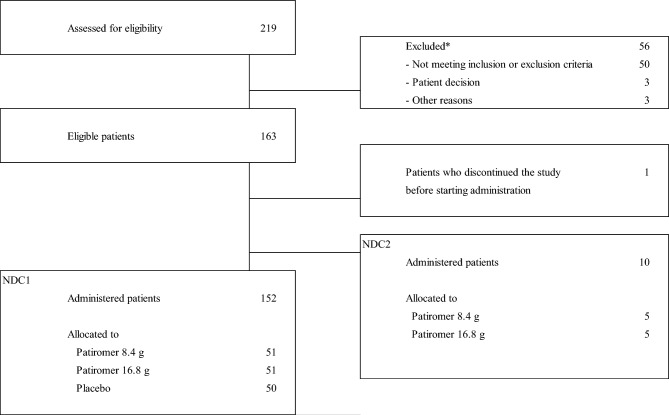
Disposition of patients in NDC1 and NDC2 by starting dose.*The total number of excluded patients is presented. More than one reason for screening failure were reported in some patients

Initially, 266 patients were screened and 185 patients were randomized; 153 patients entered NDC1, 10 patients entered NDC2, and, 22 patients entered DC (NDC1 and NDC2; Fig. 2, DC: Supplementary Fig. 1). In NDC1, one patient discontinued before receiving treatment due to inappropriate measurement of potassium, and FAS consisted of 152 patients; 8.4 g group (*n* = 51), 16.8 g group (*n* = 51) and placebo group (*n* = 50). In NDC2 and DC, FAS consisted of 8.4 g group (*n* = 5), 16.8 g group (*n* = 5) and 8.4 g group (*n* = 11), 16.8 g group (*n* = 11), respectively. Finally, 126 patients completed the study (NDC1, *n* = 107; NDC2, *n* = 5; DC, *n* = 14) and 59 discontinued. The reasons for discontinuation were shown in Supplementary Table 4. In NDC1, one patient discontinued by Week 1 due to patient decision in the 8.4 g group and none in the 16.8 g nor placebo group.

The baseline characteristics were generally well-balanced within each cohort, categorized by starting-dose group (NDC1: Table 1; NDC2 and DC: Supplementary Tables 5 and 6).

**Table 1:** Baseline characteristics by starting dose in NDC1

Characteristic		No. of patients (%)		
		Patiromer 8.4 g (*n* = 51)	Patiromer 16.8 g (*n* = 51)	Placebo (*n* = 50)
*Sex*				
Male		28 (54.9)	30 (58.8)	32 (64.0)
Female		23 (45.1)	21 (41.2)	18 (36.0)
*Age* (*years*)				
Mean		70.1	66.9	67.9
SD		7.4	8.9	9.1
< 65		11 (21.6)	17 (33.3)	12 (24.0)
> = 65		40 (78.4)	34 (66.7)	38 (76.0)
*Body mass index *(kg/m^2^)				
Mean		24.41	24.64	24.98
SD		3.44	3.79	3.25
*Central serum potassium at baseline* (mmol/L)				
Mean		5.39	5.31	5.35
SD		0.40	0.37	0.40
< 5.1		10 (19.6)	11 (21.6)	11 (22.0)
5.1 to < 5.5		21 (41.2)	24 (47.1)	17 (34.0)
5.5 to < 6.0		15 (29.4)	13 (25.5)	19 (38.0)
> = 6.0		5 (9.8)	3 (5.9)	3 (6.0)
*Stage of chronic kidney disease*				
1		0 (0.0)	1 (2.0)	1 (2.0)
2		2 (4.0)	3 (5.9)	5 (10.0)
3a		7 (14.0)	7 (13.7)	6 (12.0)
3b		14 (28.0)	12 (23.5)	6 (12.0)
4		18 (36.0)	23 (45.1)	20 (40.0)
5		9 (18.0)	5 (9.8)	12 (24.0)
*Concomitant diseases and conditions*				
Diabetes mellitus		35 (68.6)	33 (64.7)	35 (70.0)
Heart failure		6 (11.8)	6 (11.8)	6 (12.0)
Hypertension		50 (98.0)	49 (96.1)	46 (92.0)
RAASi use		39 (76.5)	41 (80.4)	39 (78.0)

Abbreviations: *DM* diabetes mellitus, *HF* heart failure, *RAASi* renin–angiotensin–aldosterone system inhibitor

The mean (± SD) daily doses during the study for the 8.4 g group were 13.38 ± 5.68 g/day in NDC1 and NDC2, 11.97 ± 6.38 g/day in DC, those for the 16.8 g group were 15.68 ± 4.93 g/day in NDC1 and NDC2, 12.45 ± 4.25 g/day in DC (data not shown).

The mean compliance proportion was 97.35%, 99.04% and 95.95% in NDC1, NDC2 and DC, respectively (data not shown).

Table 1 Baseline characteristics by starting dose in NDC1.

### Efficacy

**Fig. 3:**
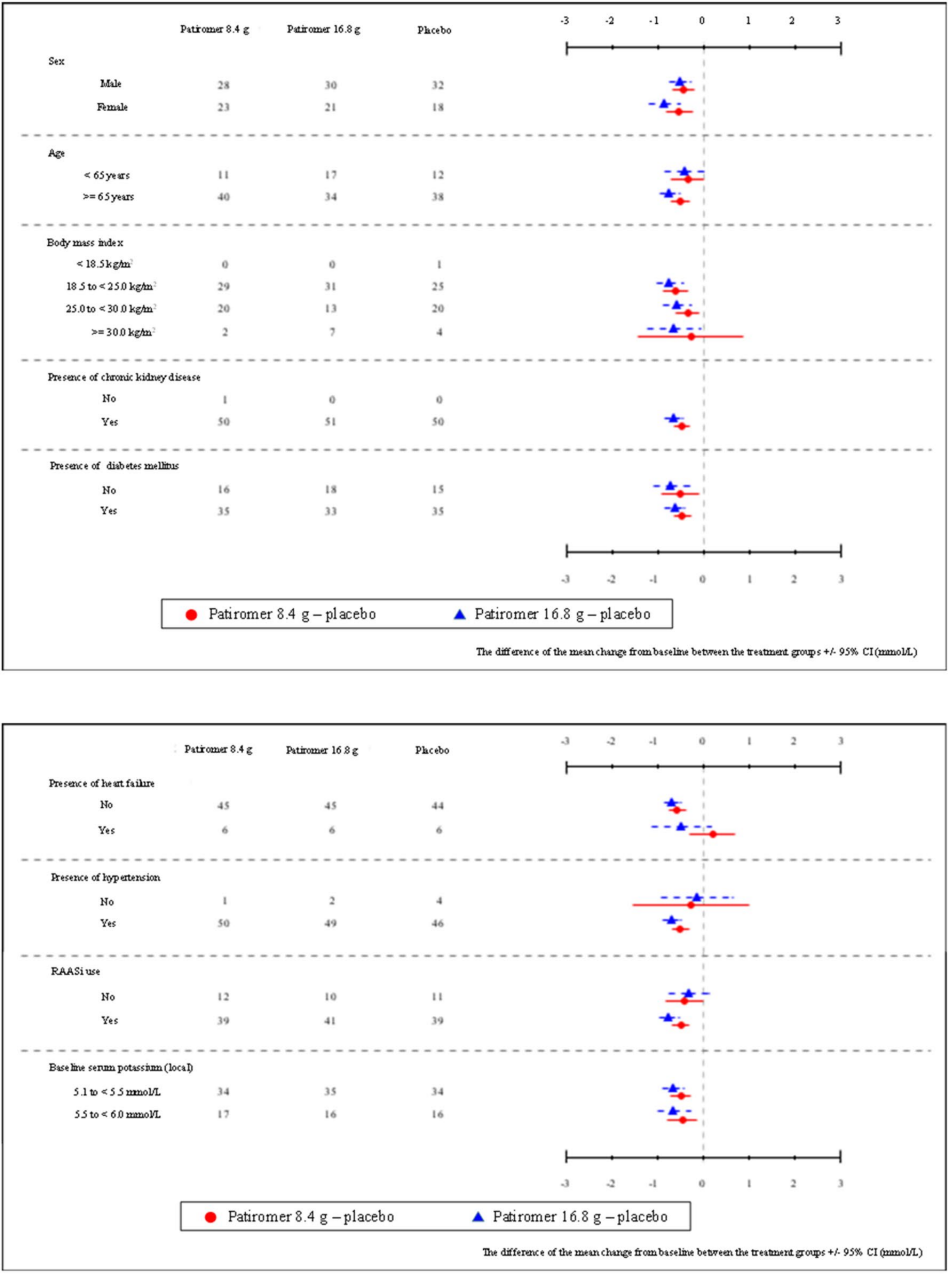
Forest plots of the subgroup analysis at Week 1 by starting dose in NDC1

The primary endpoint of the change in least squares mean sK levels at Week 1 in NDC1 was -0.55 mmol/L (95%CI,  – 0.67 to  – 0.43) for the 8.4 g group,  – 0.77 ( – 0.89 to  – 0.64) for the 16.8 g group and  – 0.10 ( – 0.22 to 0.01) for the placebo group (*P* < 0.001 for comparison between patiromer group and placebo group). A significant reduction was observed in each patiromer group compared to the placebo group (*P* < 0.001). In addition, the 16.8 g group exhibited a significant reduction compared to the 8.4 g group (*P* = 0.009) (Table 2). Mean sK change at other visits in NDC1 is shown in Supplementary Table 7. Forest plots of the subgroup analyses in NDC1 are shown in Fig. 3. Mean sK change at Week 1 was similar in all subgroups by each patiromer group. The proportion of normokalemic patients at Week 1 in NDC1 was 70.6% for the 8.4 g group, 84.3% for the 16.8 g group, and 36.0% for the placebo group (Table 3).

**Table 2:** Change from baseline in serum potassium at Week 1 by starting dose in NDC1 (analysis of covariance)

Treatment group	Change from baseline serum potassium (mmol/L)						ANCOVA		
	*n*	Mean	SD	Min	Median	Max	LSMean and 95%CI		Contrast test
Patiromer 8.4 g	51	– 0.58	0.51	– 2.0	– 0.60	0.3	– 0.55 (– 0.67, – 0.43)		*t* = – 7.464
Patiromer 16.8 g	51	– 0.75	0.56	– 2.5	– 0.80	0.4	– 0.77 (– 0.89, – 0.64)		*P* < 0.001
Placebo	50	– 0.10	0.39	– 1.0	– 0.05	0.8	– 0.10 (– 0.22, 0.01)		

Change from baseline in Serum Potassium (mmol/L)						
ANCOVA with two groups						
(i)—(j)	LSMean and 95%CI (i)	LSMean and 95%CI (j)	Difference of LSMean and 95%CI			
Patiromer 8.4 g–placebo	– 0.57 (– 0.68, – 0.45)	– 0.11 (– 0.22, 0.00)	– 0.46 (– 0.61, -0.29)		F = 30.884	*P* < 0.001
Patiromer 16.8 g–placebo	– 0.76 (– 0.88, -0.63)	– 0.09 (– 0.21, 0.03)	– 0.66 (– 0.83, – 0.48)		F = 56.278	*P* < 0.001
Patiromer 16.8 g–Patiromer 8.4 g	– 0.78 (– 0.89, -0.65)	– 0.55 (– 0.66, – 0.42)	– 0.23 (– 0.40, – 0.05)		F = 7.026	*P* = 0.009

Analysis of covariance (ANCOVA) with each treatment group as explanatory variable and baseline serum potassium as covariate

*LSMean* Least Square Mean

Difference of LSMean = each patiromer group—placebo group, patiromer 16.8 g group—patiromer 8.4 g group

Contrast test: Contrast coefficient is “patiromer 8.4 g: patiromer 16.8 g: placebo = 1:1:-2”

**Table 3:** Proportion of normokalemic (3.8–5.0 mmol/L) patients by starting dose in NDC1

Visit	Treatment group	n	Normalization	Non-normalization	Proportion of normokalemic patients (95%CI)
Week1	Patiromer 8.4 g	51	36	15	70.6 (56.2, 82.5)
	16.8 g	51	43	8	84.3 (71.4, 93.0)
	Placebo	50	18	32	36.0 (22.9, 50.8)
Week2	Patiromer 8.4 g	50	39	11	78.0 (64.0, 88.5)
	16.8 g	51	44	7	86.3 (73.7, 94.3)
	Placebo	50	37	13	74.0 (59.7, 85.4)
Week3	Patiromer 8.4 g	48	42	6	87.5 (74.8, 95.3)
	16.8 g	50	39	11	78.0 (64.0, 88.5)
	Placebo	50	40	10	80.0 (66.3, 90.0)
Week4	Patiromer 8.4 g	48	45	3	93.8 (82.8, 98.7)
	16.8 g	48	40	8	83.3 (69.8, 92.5)
	Placebo	47	41	6	87.2 (74.3, 95.2)
Week5	Patiromer 8.4 g	48	43	5	89.6 (77.3, 96.5)
	16.8 g	47	42	5	89.4 (76.9, 96.5)
	Placebo	45	37	8	82.2 (68.0, 92.0)
Week9	Patiromer 8.4 g	46	39	7	84.8 (71.1, 93.7)
	16.8 g	46	37	9	80.4 (66.1, 90.6)
	Placebo	41	39	2	95.1 (83.5, 99.4)
Week29	Patiromer 8.4 g	41	37	4	90.2 (76.9, 97.3)
	16.8 g	42	38	4	90.5 (77.4, 97.3)
	Placebo	36	33	3	91.7 (77.5, 98.3)
Week53	Patiromer 8.4 g	37	31	6	83.8 (68.0, 93.8)
	16.8 g	37	32	5	86.5 (71.2, 95.5)
	Placebo	33	29	4	87.9 (71.8, 96.6)

Table 2 Change from baseline in serum potassium at Week 1 by starting dose in NDC1 (analysis of covariance).

Table 3 Proportion of normokalemic (3.8–5.0 mmol/L) patients by starting dose in NDC1.

In NDC2, the mean sK change at Week 1 was -0.66 mmol/L for the 8.4 g group, and  – 0.86 mmol/L for the 16.8 g group (Supplementary Table 8). The proportion of normokalemic patients at Week 1 was 20.0% for the 8.4 g group, and 60.0% for the 16.8 g group (Supplementary Table 9).

In DC, the mean sK change at Week 1 was  – 0.66 mmol/L for the 8.4 g group, and  – 1.25 mmol/L for the 16.8 g group (Supplementary Table 10). The proportion of normokalemic patients at Week 1 was 72.7% for the 8.4 g group and 100.0% for the 16.8 g group (Supplementary Table 11).

**Fig. 4:**
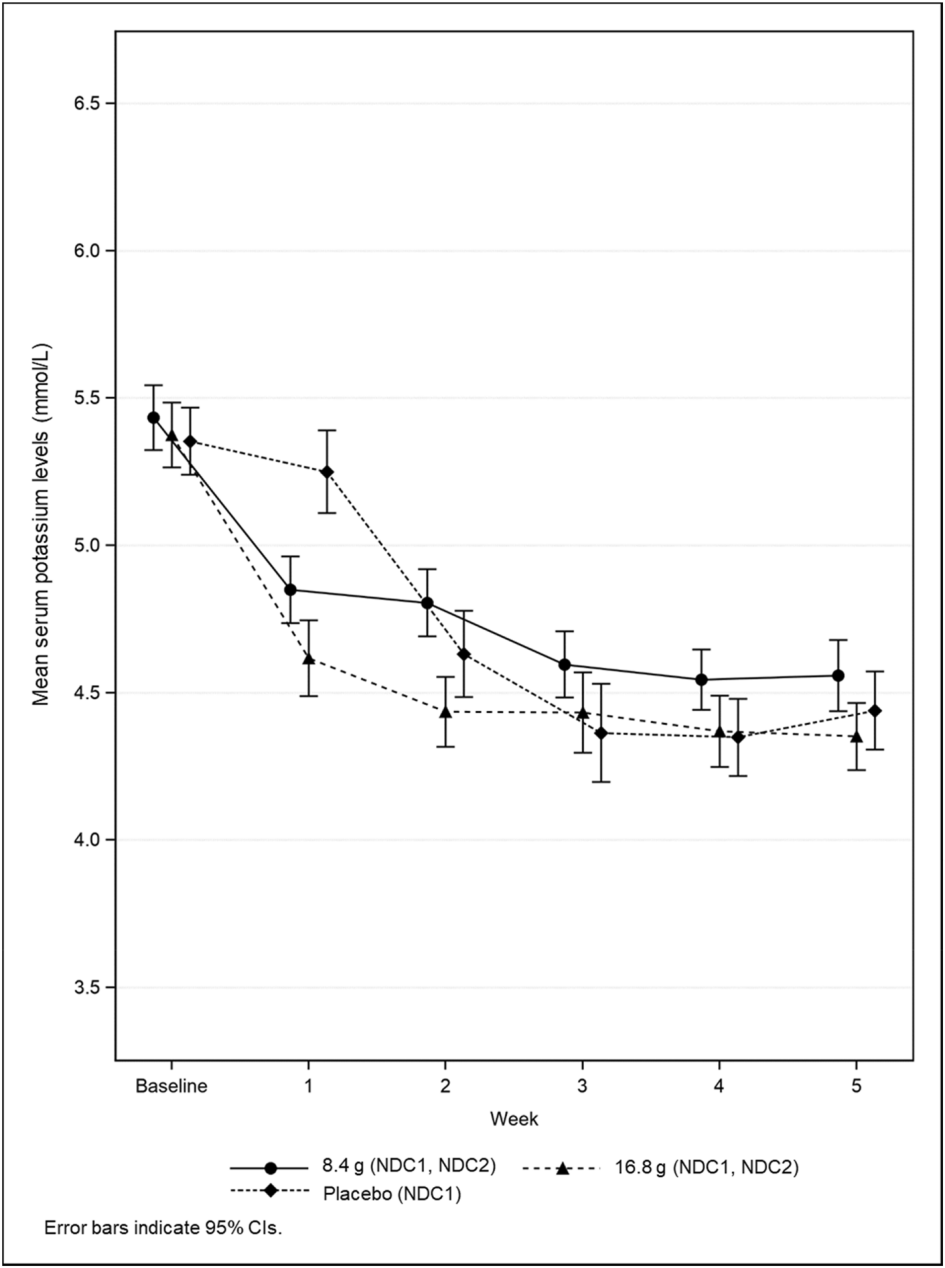
Mean serum potassium levels up to Week 5 by starting dose in NDC1 and NDC2

**Fig. 5:**
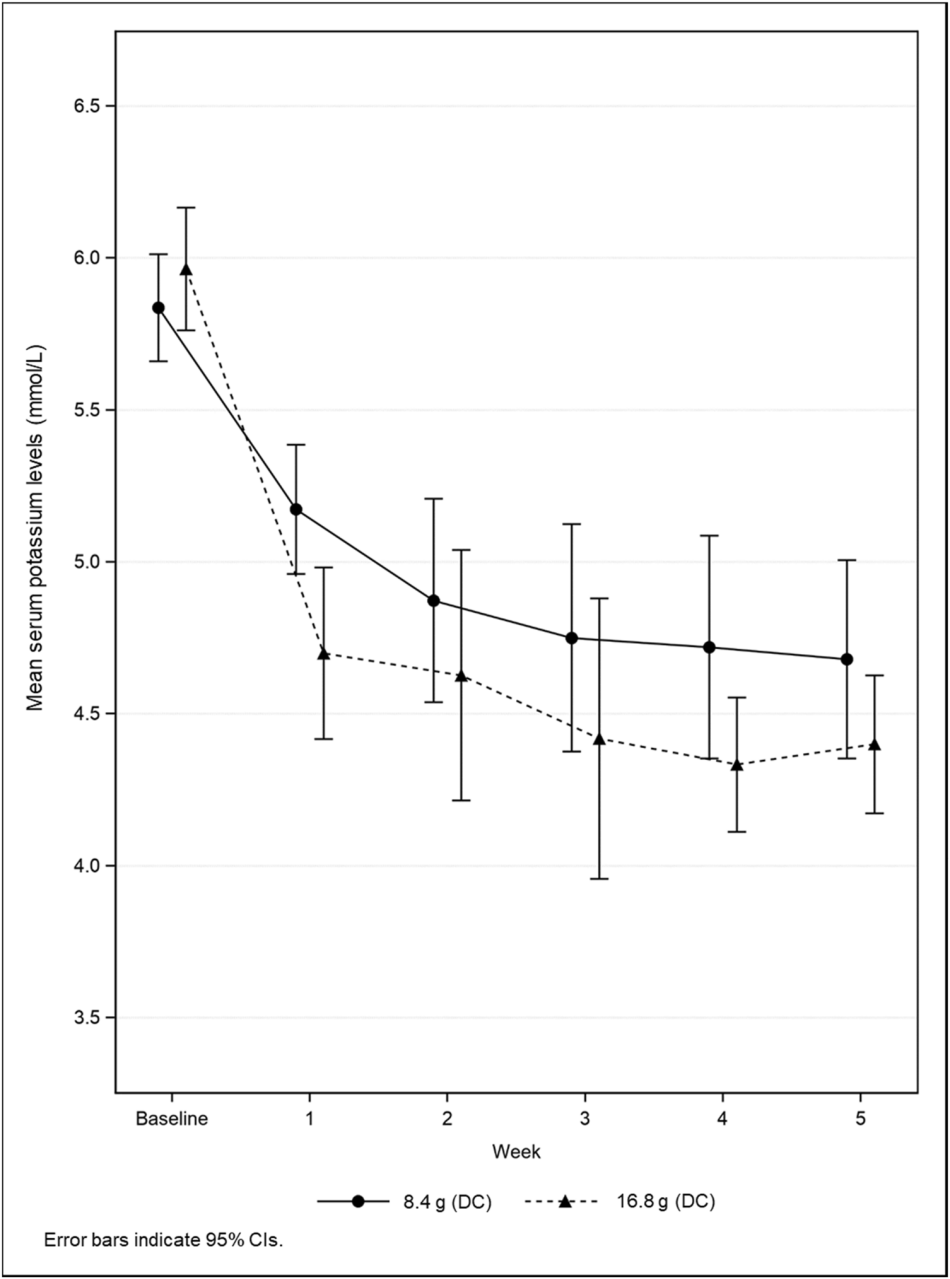
Mean serum potassium levels up to Week 5 by starting dose in DC

**Fig. 6:**
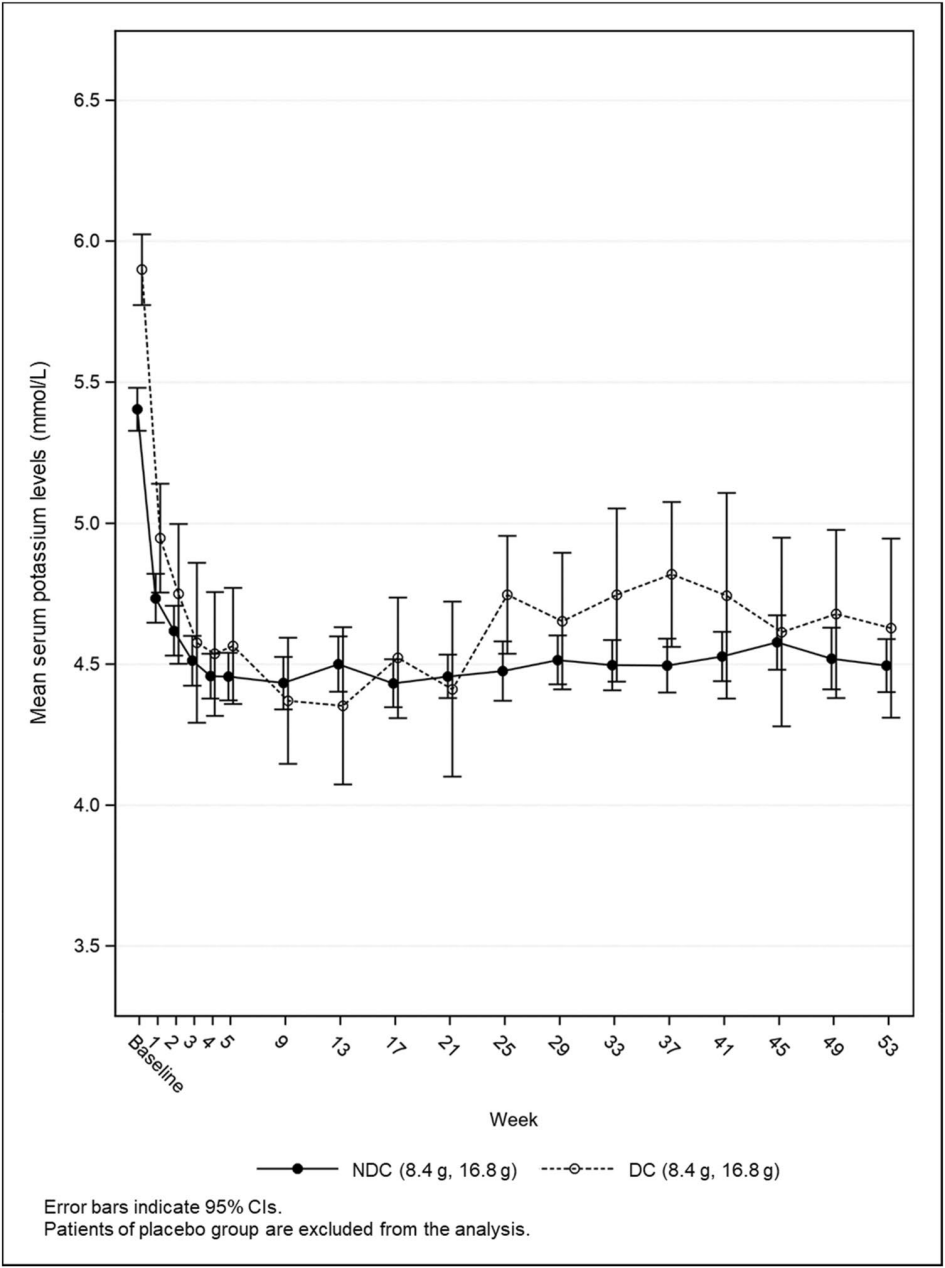
Mean serum potassium levels over 53 weeks in NDC1 and NDC2, DC

The mean sK levels until Week 5 by starting dose group are shown in Figs. 4, 5 and those throughout the treatment period are shown in Fig. 6. Regardless of cohort, mean sK levels were in the normal range at every time point after Week 5. The proportion of normokalemic patients in the patiromer group at each visit between Week 9 and 53 ranged from 81.3% to 91.8% in NDC1 and NDC2, and from 82.4% to 100.0% in DC; 84.8% in NDC1 and NDC2 and 92.9% in DC at Week 53 (Supplementary Table 12). The results of efficacy endpoints in subgroup were consistent with those in the overall population (data not shown).

In 140 patients from all cohorts who had been receiving RAASi at screening, 129 patients (92.1%) continued receiving RAASi during the study (data not shown).

### Safety

Safety was evaluated in 184 patients (NDC1, NDC2 and DC) and patiromer was generally well tolerated over the 53 weeks of treatment.

Adverse events were experienced in 160 patients (87.0%), with treatment-related adverse events being observed in 68 patients (37.0%). Serious adverse events were experienced in 31 patients (16.8%) (Supplementary Table 13); none of them was recognized as a drug-related event by investigators (attending physicians). Two deaths occurred due to lung neoplasm malignant and brain stem haemorrhage. The most frequent adverse event was constipation (26.6%) (Table 4). Most cases of constipation occurred by Week 5 and its frequency for the 16.8-g group was higher than that for the 8.4-g group especially at Week 1. All cases of constipation were mild to moderate, however, two patients discontinued the treatment because of their intolerability. In this study from all cohorts, seven patients had edema, three patients had cardiac failure, two patients had cardiac failure congestive and one patient had cardiac failure acute, respectively, and none of them was recognized as a drug related event by investigators (attending physicians). All treatment-related adverse events by Week 1 in NDC1 was shown in Supplementary Table 14.

**Table 4:** Common adverse events during the study

Adverse event^a^	*n* = 184	
	*n*	(%)
Constipation	49	(26.6)
Nasopharyngitis	44	(23.9)
Diarrhoea	13	(7.1)
Diabetes mellitus	10	(5.4)
Back pain	10	(5.4)
Chronic kidney disease	10	(5.4)

^a^Occurring in 5.0% or more of patients

Table 4 Common adverse events during the study.

Over this study in all cohorts, 18 patients (9.8%) had at least one sK level < 3.5 mmol/L; of these, the adverse event of hypokalemia was observed in one patient and no patients developed sK < 2.5 mmol/L. In each starting dose group from all cohorts, at least one sK level < 3.5 mmol/L was observed in five patients in the 8.4-g group, seven patients in the 16.8-g group, and six patients in the placebo group. Of these in the placebo group, five patients started from patiromer 16.8 g, and one patient from 25.2 g at Week 1 (data not shown). This indicates that more patients who started patiromer treatment from 16.8 g or 25.2 g than patients with 8.4 g had at least one sK level < 3.5 mmol/L.

Mean serum magnesium, calcium and phosphorus levels remained within normal range during the study (Supplementary Tables 15, 16, 17). Subgroup by serum phosphorus (sP) level at baseline was evaluated in post hoc analysis. In patients with sP level at baseline ≥ 4.6 mg/dL, mean (± SD) change from baseline in the patiromer group at Week 53 was  – 0.54 ± 0.53 mg/dL in NDC1 and NDC2, and -0.71 ± 1.16 mg/dL in DC. Mean (± SD) serum bicarbonate for the patiromer group at baseline and Week 53 was 21.20 ± 3.48 mmol/L and 19.79 ± 4.11 mmol/L in NDC1, 19.63 ± 3.30 mmol/L and 18.76 ± 4.81 mmol/L in NDC2, 18.76 ± 2.87 mmol/L and 16.89 ± 2.18 mmol/L in DC.

## Discussion

In this phase two, placebo controlled and long-term study, patiromer showed significant reduction of sK levels compared to placebo at Week 1 in NDC1, with consistent potassium-lowering effects observed over 53 weeks and high adherence across the all cohorts. There was no serious adverse event considered treatment-related by investigators and the safety profile of patiromer was preserved in this study.

These results ascertained the clinical benefits of patiromer for Japanese patients with hyperkalemia, including those undergoing dialysis. The 8.4 g starting dose of patiromer significantly reduced sK levels compared to placebo at Week 1 in NDC1. Most patients in the 8.4 g group in NDC1 achieved normal sK levels at Week 1 and proportions of normokalemic patients in the 8.4-g group were similar to those in the 16.8 g group after Week 5 across the all cohorts. This indicates that sK level is well controlled with the treatment of patiromer once a day with dose titration by Week 5 regardless of starting dose. Given that the frequency of constipation was higher in the 16.8 g group at Week 1 and more patients who started patiromer treatment from 16.8 g or 25.2 g including the placebo group had a decrease in at least one sK level < 3.5 mmol/L, 8.4 g is an optimal starting dose to reduce the risk of safety concerns to minimum. Meanwhile, 16.8-g starting dose may be a preferable treatment option for patients whose sK levels are required to decrease earlier than next dose titration, considering a significant dose dependent reduction in sK levels at Week 1 in NDC1. In NDC2, proportion of normokalemic patients in the 16.8 g group was higher than that in the 8.4 g group at Week 1. This indicates that 16.8 g starting dose could be an option for moderate or greater hyperkalemia. Hyperkalemia is a major limitation to continue RAASi treatment [8, 9, 15]. During the study, 92.1% of patients continued receiving RAASi, which suggests that patiromer can contribute to keep patients on the treatment of RAASi by maintaining long-term sK control with good adherence.

Mild-to-moderate constipation, the most frequent adverse event with patiromer, were tolerable in most cases. Severe gastrointestinal side effects including bowel necrosis which have been reported with other traditional potassium binders were not observed.

Sodium polystyrene sulfonate and sodium zirconium cyclosilicate contain sodium and monitor for signs of edema is advised particularly in patients who should restrict their sodium intake or are prone to fluid overload (e.g., heart failure or CKD) [16, 17]. These diseases comprise a significant proportion of the population with or at risk for hyperkalemia. Patiromer, a novel potassium-binding polymer, contains calcium rather than sodium as the counter ion exchange, thus avoiding the risk of a sodium load in patients. No treatment-related edema or heart failure was observed in this study.

In patients with sP level at baseline ≥ 4.6 mg/dL, sP level decreased by 0.5–1.0 mg/dL. Decrease in sP level in patients with hyperphosphatemia is consistent with other clinical studies in non-Japanese patients with hyperkalemia [18, 19]. In addition, earlier clinical studies in healthy volunteers showed that patiromer increased fecal phosphorus level and decreased urine phosphorus level on a controlled diet while mean sP level was stable throughout the treatment periods [20]. These indicate that through binding to calcium ion, phosphorus absorption from the gastrointestinal tract may be decreased, resulting in the decrease of sP level in patients with hyperphosphatemia. There were no clinically relevant changes in laboratory parameters except sK.

This study has several limitations. The study population included only Japanese patients and the relatively small number of patients in DC. Placebo-controlled study design was limited to one week. Dietary potassium intake and urinary potassium excretion were not evaluated: however, patients were instructed not to change eating habits so far before and after participating in this study. Dialysis vintage was not recorded.

## Conclusions

Patiromer demonstrated a significant reduction in sK levels, with a notable dose-dependent reduction observed at Week 1. Treatment with patiromer was effective in lowering and maintaining sK levels, with a good safety profile over 53 weeks in Japanese patients with hyperkalemia.

Results of this study suggest that patiromer will be a favorable long-term treatment option in Japanese patients with hyperkalemia, with the 8.4 g dose identified as an optimal starting point to minimize safety concerns.

Further confirmatory studies are needed to verify the efficacy of patiromer in Japanese patients with hyperkalemia.

## Supplementary Information

Below is the link to the electronic supplementary material.

Supplementary file1 (DOCX 88 KB)
