# Supplementary material for: Efficacy and safety of patiromer for non-dialysis and dialysis patients with hyperkalemia: the randomized, placebo-controlled and long-term study
Source: Clin Exp Nephrol. 2024 Nov 24;29(5):548–59. doi: 10.1007/s10157-024-02585-5 (PMC12049377; doi:10.1007/s10157-024-02585-5)
Supplement: Supplementary file 1 — Supplementary file1 (DOCX 88 KB) [file 10157_2024_2585_MOESM1_ESM.docx]

**Supplementary Material**

Supplementary Table 1. Additional details for allocation

Supplementary Table 2. List of potassium-altering chronic medications

Supplementary Table 3. Analysis population

Supplementary Table 4. Reasons for discontinuation by cohort and starting dose

Supplementary Table 5. Baseline characteristics by starting dose (NDC2)

Supplementary Table 6. Baseline characteristics by starting dose (DC)

Supplementary Table 7. Change from baseline in serum potassium by starting dose in NDC1

Supplementary Table 8. Change from baseline in serum potassium by starting dose in NDC2

Supplementary Table 9. Proportion of normokalemic (3.8-5.0 mmol/L) patients in NDC2

Supplementary Table 10. Change from baseline in serum potassium in DC

Supplementary Table 11. Proportion of normokalemic (3.8-5.5 mmol/L) patients in DC

Supplementary Table 12. Proportion of normokalemic (3.8-5.0 mmol/L) patients in NDC1 and NDC2

**Supplementary Table 13. Serious adverse events by cohort**

**Supplementary Table 14. All treatment-related adverse events by Week 1 in NDC**

**Supplementary Table 15. Summary statistics of serum magnesium (mg/dL)**

**Supplementary Table 16. Summary statistics of serum calcium (mg/dL)**

**Supplementary Table 17. Summary statistics of serum phosphorus (mg/dL)**

Supplementary Figure 1. Disposition of patients in DC.

*The total number of excluded patients is presented. More than one reason for screening failure might be reported for a patient.
